# Increased Risk of Coronary Artery Disease in People with a Previous Diagnosis of Carpal Tunnel Syndrome: A Nationwide Retrospective Population-Based Case-Control Study

**DOI:** 10.1155/2019/3171925

**Published:** 2019-03-03

**Authors:** Yi-Chuan Chang, Jen-Huai Chiang, Ing-Shiow Lay, Yu-Chen Lee

**Affiliations:** ^1^Department of Chinese Medicine, China Medical University Beigang Hospital, Yunlin 601, Taiwan; ^2^Graduate Institute of Acupuncture Science, China Medical University, Taichung 404, Taiwan; ^3^Management Office for Health Data, China Medical University Hospital, Taichung 404, Taiwan; ^4^College of Medicine, China Medical University, Taichung 404, Taiwan; ^5^Department of Chinese Medicine, China Medical University Hospital, Taichung 404, Taiwan

## Abstract

**Background:**

Existing literature suggests that an association exists between coronary artery disease (CAD) and carpal tunnel syndrome (CTS), but few researchers have explored whether CTS is a risk for CAD.

**Materials and Methods:**

This large case-control study explored the association between CTS and CAD, using health insurance claims data from Taiwan's National Health Insurance Research Database (NHIRD) between January 2000 and December 2013. International Classification of Diseases, Ninth Revision, Clinical Modification (ICD-9-CM) diagnostic codes identified 70,622 patients with CAD (ICD-9-CM codes 410–414; cases); 70,622 patients without CAD were frequency-matched by age, sex, and index year of CAD and served as controls.

**Results:**

The likelihood of a previous diagnosis of CTS (ICD-9-CM codes 354.0, 354.1) was significantly higher in the CAD group than in the comparison control group (crude OR: 1.75, 95% CI 1.63-1.89; p<0.0001; adjusted OR: 1.46, 95% CI 1.34-1.58; p<0.0001).

**Conclusion:**

A significant positive correlation was observed between CAD and a previous diagnosis of CTS.

## 1. Introduction

Coronary artery disease (CAD) is caused by a thickening of the inner walls of the coronary arteries. Narrowing of the vessel space, particularly of the arteries, decreases blood flow and can lead to CAD symptoms or even death. The prevalence of CAD markedly increases with age [1], as is illustrated by long-term follow-up from the Framingham Study, in which the incidence of CAD between the ages of 65 and 94 years was more than doubled in males and tripled in females compared to individuals aged 35–64 years [2]. Hyperlipidemia (excessively high blood levels of low-density lipoprotein (LDL) cholesterol) is a risk factor for inducing inflammation in vessels [3]. Inflammation plays an important role in chronic diseases such as hypertension and diabetes mellitus (DM), both of which are associated with an increased risk of CAD. It is speculated that reductions of 10–12 mmHg in systolic pressure and of 5–6 mmHg in diastolic pressure may reduce the risk of CAD by 16% [4], while other research indicates that people with DM have double the risk of developing CAD compared with people without DM [5].

Carpal tunnel syndrome (CTS) is characterized by tingling in the fingers innervated by the median nerve, due to its compression in the carpal tunnel [6–8]. Edema, tendon inflammation, hormonal changes and manual activity can contribute to nerve compression [9]. An impaired vascular supply may increase the vulnerability of the nerve to mechanical loading and prolonged tissue ischemia can lead to degeneration of the nerve and intraneural fibrosis [10, 11]. Some conditions that may increase the vulnerability of the median nerve include DM [12–14], rheumatoid arthritis [15], smoking [16–18], and alcohol-related toxic effects [18], as well as high physical workload exposure [19].

Recent data indicate that the severity of CTS correlates with body mass index (BMI) and waist circumference [20], and that each 1-unit increase in BMI increases the risk of CTS by 7.4% [21]. Individuals with metabolic syndrome are more likely to have a higher BMI than normal blood pressure or serum lipid levels [22], which are associated with CAD [3–5], but metabolic syndrome problems for atherosclerotic risk factors also effect in CTS with evidence supports [23–25]. One study suggests that an association exists between CTS and cardiovascular risk factors in middle-aged people (aged 30–44 years), while CTS appears to be associated with carotid artery intima-media thickness (IMT) and clinical atherosclerotic vascular disease in older-aged persons (aged ≥60 years) [22]. It has been suggested that CTS may be a manifestation of atherosclerosis, or that these conditions share similar risk factors [22].

Occupation-related investigations have suggested a strong relationship between CAD and CTS [26], but some data have indicated that cardiovascular risk factors or the metabolic syndrome may increase the risk of CTS [26, 27]. Our study aimed to determine the risk of CAD in people with a previous diagnosis of CTS. We hypothesized that CTS could be a predictive factor for developing CAD, even without the involvement of occupational-related variables.

## 2. Materials and Methods

### 2.1. Data Resources

This case-control study used health insurance claims data from Taiwan's National Health Insurance Research Database (NHIRD). The NHIRD records are sourced from reimbursement claims covering more than 99% of the total population of Taiwan and contain demographic information, records of clinical visits, hospitalizations, diagnoses, assessments, procedures, prescriptions, and medical costs for reimbursement. Analyses of NHIRD data enable researchers to examine potential associations between diseases, track the clinical development of diseases, and explore outcomes of various medical interventions. This unique, nationwide database is providing researchers with a large repository of clinical records that are proving valuable for examinations into many different avenues of research [28–30].

Our data were obtained from the Longitudinal Health Insurance Database 2000 (LHID2000), a subset of the NHIRD consisting of a random sample of longitudinally linked data from 1996 through 2013 for 1 million individuals. All of the data were deidentified and encrypted prior to release for publication, to prevent any possible identification of individuals. Patients with CAD and CTS were identified by the International Classification of Diseases, Ninth Revision, Clinical Modification (ICD-9-CM) diagnostic codes. Confirmation of these diagnoses required that they were cited at least 3 times on different dates, as a means of preventing inclusion of subjects who were misdiagnosed or whose diagnostic codes changed following clinical findings or medical examinations [31].

### 2.2. Study Subjects and Study Variables

**Figure 1:**
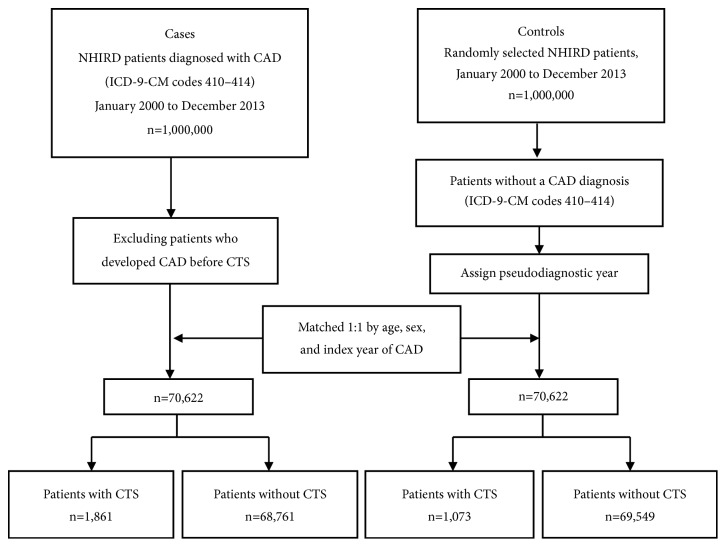
Flow chart of subjects from the LHID 2000 for patients with CAD (cases) and those without CAD (controls).

The analysis included 70,622 cases with newly diagnosed CAD (ICD-9-CM codes 410-414) within the study period and 70,622 individuals without CAD (controls) randomly selected from the same database and frequency-matched to cases by age (within 5 years), sex and index year of CAD. All subjects were screened for this study from NHIRD records between January 2000 and the end of 2013 and followed to death, or the occurrence of a CTS diagnosis (ICD-9-CM codes 354.0, 354.1) previously in individual-level data. For each CAD case, the index year of CAD was defined as the year of the initial diagnosis of CAD; for the controls, the index year of CAD was randomly assigned from January 2000 to December 2013, according to the index year of the matched CAD case. Patients with records reporting a history of DM (ICD-9-CM code 250), hypertension (ICD-9-CM codes 401–405), or hyperlipidemia (ICD-9-CM code 272) at the index date were considered to have comorbidities. In order to focus on the effect and causality between CAD and CTS, subjects with CAD occurring before CTS were excluded from analysis (Figure 1).

### 2.3. Statistical Analysis

All statistical analyses were performed with SAS® statistical analysis software, version 9.4 (SAS Institute Inc., Cary, NC, USA). Descriptive statistics examined the baseline characteristics between cases and controls. The Pearson's chi-squared test was applied for categorical variables (e.g., sex) and the Student's* t*-test for continuous variables (e.g., age). We used logistic regression analysis to estimate odds ratios (ORs) and 95% confidence intervals (CIs) for each variable. Multivariate analysis adjusted for sociodemographic factors and comorbidities. All data are reported as the mean with one standard deviation (SD) in parentheses or as rates with 95% CIs. A* P* value of <0.05 was considered to be statistically significant.

## 3. Results

Table 1 shows the demographic and clinical characteristics of the study population. After excluding those individuals who were diagnosed with CAD before developing CTS, each study population included 70,622 patients with or without CAD. A prior diagnosis of CTS was identified in 1,861 (2.64%) of the cases and in 1,073 (1.52%) of the controls. The sex and age distributions were similar between the groups; DM, hypertension, and hyperlipidemia were all significantly more prevalent among the cases compared with controls. In each study group, most patients were aged between 40 and 65 years.

Odds ratios and confidence intervals for the study population are depicted in Table 2. CTS diagnoses were significantly more likely in the CAD group than in controls (crude OR, 1.75; 95% CI, 1.63-1.89;* P *<0.0001). This association persisted in logistic regression modeling adjusting for the potentially confounding variables of sex, age, DM, hypertension, and hyperlipidemia (adjusted OR, 1.46, 95% CI 1.34-1.58;* P* <0.0001).

As shown in Table 3, in logistic regression analysis incorporating sex, age, and comorbidities, patients with CAD were significantly more likely than controls to have a previous diagnosis of CTS; all* P* values were <.05, even after adjusting for the confounding variables of sex, age, and comorbidities.

## 4. Discussion

This analysis of NHIRD data supports our contention that patients with a previous diagnosis of CTS are at significant risk of developing CAD (*P *<0.0001). Existing evidence supports an association between CAD and the risk factors of hyperlipidemia, DM and hypertension [3, 4], while more recent research also suggests an association between some neuromuscular diseases, including CTS, as risk factors for CAD [26]. That research is supported by our study findings indicating that a previous diagnosis of CTS could be a risk factor for CAD. Interestingly, a study including 6,254 patients reported associations between cardiovascular risk factors of obesity, dyslipidemia and hypertension and CTS in patients aged 30–44 years, while in those aged ≥60 years, CTS was associated with CAD and carotid IMT [22]. The association between CTS and carotid IMT was strong evident in patients exposed to physical load factors and in those with hypertension or atherosclerotic disease [22]. Other researchers have also reported finding a significantly increased prevalence of carotid IMT in patients with CTS [32, 33].

Chronic inflammation affecting almost all stages (initiation, growth, and destabilization) of atherosclerotic lesions is a characteristic feature of atherosclerosis and lead to CAD [34, 35]. Inflammation also plays an important role in carotid IMT [36, 37]. DM and hypercholesterolemia influence the association between carotid IMT and C-reactive protein, a biomarker of inflammation [37]. Moreover, blood pressure and hypercholesterolemia appear to influence the association between carotid IMT and fibrinogen [38]. These data suggest possible mechanisms and pathological associations connecting CTS with CAD, DM, hypertension and hyperlipidemia; chronic inflammation beyond the traditional cardiovascular risk factors might be related to increased carotid IMT observed in patients with CTS [32].

Our study makes three important contributions. First, the validity of our results is strengthened by the use of a large, representative, nationwide, general population-based database to survey the risk of CAD in patients with a previous diagnosis of CTS. Second, we used those NHIRD records to investigate time sequences for the possible association between CTS and CAD. We identified an increased incidence of CAD in individuals with a previous diagnosis of CTS, suggesting that there may be a cause and effect relationship between CTS and the later development the more severe, complicated medical disorder of CAD. Third, repetitive hand movements or excessive work loading of the hand and wrist are considered to be risk factors for CTS, especially in certain populations such as industrial workers [19, 26, 39], although our study findings suggest that CAD may develop in individuals with a previous diagnosis of CTS with no involvement of occupational variables.

Limitations of our study include the fact that it was based on NHIRD data; first, it was impossible to ascertain levels of disease severity and variation using the ICD-9-CM codes. Although all medical records were included for every study participant, it was difficult to clarify any disease variables for analysis beyond the recorded comorbidities of DM, hypertension and hyperlipidemia. It is possible that some cases may have experienced CTS in both hands or with associated complications, but this is not able to be clarified from the information given in the NHIRD database. We therefore screened subjects for inclusion or exclusion in this study by considering similar variables in individual with CTS, rather than by affected hands. Besides the recognized association between the variables of DM, hypertension and hyperlipidemia with CAD, factors such as occupation, smoking and alcohol use, lifestyle behaviors, BMI, and hereditary conditions that affect the prognosis of CAD also play an important role and are not necessarily available for analysis in the NHIRD data. Second, while CTS has been associated with demanding sorts of physical work tasks, especially vibrating tools and those requiring high hand grip strength [39], some of the evidence is contradictory [17]. Similarly, NHIRD records do not enable insights into the strength of hand activity and repeated hand movements contributing to CTS. Moreover, renal disease, especially in hemodialysis patients, reportedly increases vulnerability to CTS [40], an aspect that was not considered in this study. Third, in respect to our research design, this study specified a narrow time range for capturing subjects and therefore is likely to underestimate the incidence of CAD; increasing our time range by approximately another 10 years would be likely to yield more occurrences of CAD as the study population ages. Importantly, it is likely that matching to within 5 years in a comparatively broad band was a source of residual confounding. Moreover, the 1:1 case-control matching used in this study increases confounding and residual confounding risks because of the small sample size of controls, which also means that the analyses are unable to make sex-related links to CAD and CTS, because subjects were matched by sex. Another important consideration is that the evidence derived from a retrospective NHIRD database is generally lower in statistical quality than evidence from randomized trials, because of potential bias in selection of subjects or bias resulting from unknown confounders. In addition, our study design did not enable us to estimate risks associated with sex from NHIRD records, such as information on menopause among the female population; it is well known that the occurrence of menopause is linked to a higher risk for CTS in old women [41]. Further research is needed to analyze data between and within each sex, as well as examine differences between age groups in each sex. Moreover, whether our results can be extrapolated to Western or non-Taiwanese populations are uncertain and it would be one of the limitations in the study.

Furthermore, while this study found an increased risk of CAD in patients with a previous diagnosis of CTS, it remains unclear as to whether other neuromuscular diseases or symptoms may also be related to CAD. Previous research has confirmed that rotator cuff tendinopathy [42] and lateral epicondylitis [43] are related to the development of CAD, although it remains uncertain as to whether the mechanisms leading to the development of CAD are similar between these neuromuscular diseases and CTS. Importantly, the scope of our research is limited by the fact that some symptoms or signs cannot be diagnosed as a disease relating to a specific ICD-9-CM code. Another important consideration is that it is unclear as to whether the NHIRD records are representative of global disease characteristics. Finally, although our data indicate an association between CAD and CTS over an approximate 10-year period, it is unclear as to whether this association would endure over a longer time period.

## 5. Conclusion

In comparison with the non-CAD group, patients with CAD were more likely to have a previous diagnosis of CTS (crude OR: 1.75, 95% CI 1.63-1.89;* P *<0.0001; adjusted OR: 1.46, 95% CI 1.34-1.58;* P *<0.0001). Further research is needed to clarify whether prior CTS is a true risk factor and/or signals increased susceptibility to CAD.

## Figures and Tables

**Table 1:** Demographic and clinical characteristics of the study population.

Variable	No CAD (controls)		CAD (cases)		*P*-value
	(n=70,622)		(n=70,622)		
	n	%	n	%	
*CTS* ^*Ɨ*^		****		****	<.0001^*∗*^
No	69,549	98.48	68,761	97.36	
Yes	1,073	1.52	1,861	2.64	
*Sex* ^*Ɨ*^					0.99
Female	33,234	47.06	33,234	47.06	
Male	37,388	52.94	37,388	52.94	
*Age, years* ^*Ɨ*^					0.9542
<18	162	0.23	152	0.22	
18–39	4,219	5.97	4,229	5.99	
40–65	39,967	56.59	39,967	56.59	
>65	26,274	37.20	26,274	37.2	
Mean ± SD^‡^	60.01±13.45		60.14±13.35		0.0677
*Comorbidity* ^*Ɨ*^					
Diabetes mellitus	10,580	14.98	18,925	26.80	<.0001^*∗*^
Hypertension	24,189	34.25	45,483	64.40	<.0001^*∗*^
Hyperlipidemia	14,069	19.92	27,638	39.14	<.0001^*∗*^

CAD = coronary artery disease; CTS = carpal tunnel syndrome; SD = standard deviation. Tests used: ^*Ɨ*^Pearson's chi-squared test; ^‡^Two-sample *t*-test. *∗P *<0.001 vs controls.

**Table 2:** Odds ratios (ORs) and 95% confidence intervals (CIs) of univariate and multivariate logistic regression analyses using patients' characteristics as predictors for the development of CAD with/without a previous diagnosis of CTS.

Variable	Crude ORs		Adjusted ORs^§^	
	(95% CI)	*P*-value	(95% CI)	*P*-value
*CTS*				
No	1 (Reference)		1 (Reference)	
Yes	1.75 (1.63-1.89)	<.0001^*∗*^	1.46 (1.34-1.58)	<.0001^*∗*^
*Sex*				
Female	1 (Reference)		1 (Reference)	
Male	1.00 (0.98-1.02)	0.99	1.09 (1.06-1.11)	<.0001^*∗*^
*Age, years*				
<18	1 (Reference)		1 (Reference)	
18–39	1.07 (0.85-1.34)	0.5657	0.84 (0.67-1.06)	0.1349
40–65	1.07 (0.85-1.33)	0.5735	0.54 (0.43-0.67)	<.0001^*∗*^
>65	1.07 (0.85-1.33)	0.5739	0.38 (0.30-0.47)	<.0001^*∗*^
*Comorbidity* ^*Ɨ*^				
Diabetes mellitus	2.08 (2.02-2.13)	<.0001^*∗*^	1.25 (1.21-1.29)	<.0001^*∗*^
Hypertension	3.47 (3.40-3.55)	<.0001^*∗*^	3.35 (3.27-3.43)	<.0001^*∗*^
Hyperlipidemia	2.58 (2.52-2.65)	<.0001^*∗*^	1.80 (1.76-2.85)	<.0001^*∗*^

^§^Adjusted for CTS, age, sex, DM, hypertension, and hyperlipidemia. ^*Ɨ*^The Reference population consisted of patients without the comorbidity. CAD = coronary artery disease; CTS = carpal tunnel syndrome. *∗ P* <0.001 vs Reference population (patients without CAD).

**Table 3:** Odds ratios (ORs) and 95% confidence intervals (CIs) of univariate and multivariate logistic regression analyses using patients' characteristics as predictors for the development of CAD with/without a previous diagnosis of CTS, with analyses stratifying for sex, age, and comorbidities.

Variable	No CTS		CTS		Crude ORs		Adjusted ORs^§^	
	No CAD	CAD	No CAD	CAD	OR (95% CI)	*P*-value	OR (95% CI)	*P*-value
	n (%)	n (%)	n (%)	n (%)				
*Sex*								
Female	32,471 (46.69)	31,924 (46.43)	763 (71.11)	1,310 (70.39)	1.75 (1.60-1.91)	<.0001^*∗∗∗*^	1.51 (1.37-1.66)	<.0001^*∗∗∗*^
Male	37,078 (53.31)	36,837 (53.57)	310 (28.89)	551 (29.61)	1.79 (1.56-2.06)	<.0001^*∗∗∗*^	1.30 (1.12-1.51)	0.0005^*∗∗*^
*Age, years*								
<18	162 (0.23)	152 (0.22)	0 (0)	0 (0)	-	-	-	-
18–39	4,204 (6.04)	4,167 (6.06)	15 (1.40)	62(3.33)	4.17 (2.37-7.34)	<.0001^*∗∗∗*^	2.82 (1.53-5.20)	0.0009^*∗∗*^
40–65	39,253 (56.44)	38,654 (56.22)	714 (66.54)	1,313 (70.55)	1.87 (1.70-2.05)	<.0001^*∗∗∗*^	1.56 (1.41-1.72)	<.0001^*∗∗∗*^
>65	25,930 (37.28)	25,788 (37.5)	344 (32.06)	486 (26.11)	1.42 (1.24-1.63)	<.0001^*∗∗∗*^	1.18 (1.02-1.36)	0.0259^*∗*^
*Comorbidity*								
*Diabetes mellitus*								
No	59,218 (85.15)	50,409 (73.31)	824 (76.79)	1,288 (69.21)	1.84 (1.68-2.01)	<.0001^*∗∗∗*^	1.52 (1.38-1.67)	<.0001^*∗∗∗*^
Yes	10,331 (14.85)	18,352 (26.69)	249 (23.21)	573 (30.79)	1.30 (1.11-1.51)	0.0008^*∗∗*^	1.20 (1.03-1.40)	0.0205^*∗*^
*Hypertension*								
No	45,868 (65.95)	24,477 (35.60)	565 (52.66)	662 (35.57)	1.73 (1.56-1.92)	<.0001^*∗∗∗*^	1.62 (1.45-1.81)	<.0001^*∗∗∗*^
Yes	23,681 (34.05)	44,284 (64.40)	508 (47.34)	1,199 (64.43)	1.20 (1.07-1.34)	0.0016^*∗∗*^	1.22 (1.09-1.37)	0.0007^*∗*^
*Hyperlipidemia*								
No	55,923 (80.41)	42,161 (61.32)	630 (58.71)	823 (44.22)	2.20 (1.96-2.46)	<.0001^*∗∗∗*^	1.81 (1.61-2.04)	<.0001^*∗∗∗*^
Yes	13,626 (19.59)	26,600 (38.68)	443 (41.29)	1,038 (55.78)	1.26 (1.14-1.40)	<.0001^*∗∗∗*^	1.13 (1.02-1.26)	0.0215^*∗∗*^

^§^Adjusted for CTS, age, sex, diabetes mellitus, hypertension, hyperlipidemia by logistic regression analysis. The Reference group consisted of patients who were not using medications for CTS. CAD = coronary artery disease; CTS = carpal tunnel syndrome. *∗P* <0.05; *∗∗P* <0.01; *∗∗∗P* <0.001; for all comparisons between subpopulations with/without CAD with a previous diagnosis of CTS.

## Data Availability

The NHIRD data used to support the findings of this study are included within the article.
